# Constraints to agroforestry diffusion under the Billion Trees Afforestation Project (BTAP), Pakistan: policy recommendations for 10-BTAP

**DOI:** 10.1007/s11356-022-20661-9

**Published:** 2022-05-12

**Authors:** Ayat Ullah, Alam Zeb, Shahab E. Saqib, Harald Kächele

**Affiliations:** 1 Department of Agriculture Extension, MNS-University of Agriculture, Multan, Pakistan; 2 Research Area 2 “Land Use and Governance”, Working group: Sustainable Land Use in Developing Countries, Leibniz Centre for Agricultural Landscape Research (ZALF), Eberswalder Straße 84, 15374 Müncheberg, Germany; 3 Department of Renewable Resources, University of Alberta, 751 General Services Building, Edmonton, AB T6G 2H1 Canada; 4 Department of Forestry, Shaheed BB University, Sheringal Dir (Upper), 18050 Khyber Pakhtunkhwa, Pakistan; 5 Department of Commerce Education and Management Sciences, Higher Education, Archives and Libraries Department, Khyber Pakhtunkhwa, Pakistan; 6 Eberswalde University for Sustainable Development, Schicklerstraße 5, 16225 Eberswalde, Germany

**Keywords:** Agroforestry diffusion, 10-Billion Trees Afforestation Project, Policy and extension, Hindu-Kush Himalayan, Village Development Committees, Planning and monitoring

## Abstract

Effective agroforestry diffusion under the newly started 10-Billion Trees Afforestation Project (10-BTAP) needs a thorough understanding of the policy and implementation shortfalls of the already completed BTAP. This study examines the factors that affected the diffusion of the agroforestry in the Hindu-Kush Himalayan (HKH) region of Pakistan under BTAP. The data were gathered through in-depth interviews with Village Development Committee (VDC) members, Forest Department (FD) officials, and local farmers. Important factors positively affecting agroforestry diffusion included locations of crop fields on the river sides, community dependency on firewood, and market value of agroforestry timber. The logistic regression model shows that household head’s age, access to information, and area under cropland positively affected household level adoption of agroforestry under BTAP; forest cover was negatively related. In-depth interviews show that key barriers to FD in diffusion of agroforestry included provision of false information by farmers to monitoring teams, non-availability of extension staff, lack of communication among project staff and community, as well as a lack of sufficient budget for activities. Primary barriers to community adoption of agroforestry included no participation of VDCs in planning and monitoring of agroforestry programs, lack of plant need assessments on the part of the project staff, poor quality of plants distributed by FD, farmers’ poor know-how of plantations, lack of trust among community and project staff, as well as the waste of plants by farming community. Thus, this study recommends that policy-makers and project designers should consider these factors when planning agroforestry diffusion under 10-BTAP to improve its success.

**Supplementary Information:**

The online version contains supplementary material available at 10.1007/s11356-022-20661-9.

## Introduction

Diffusion of agroforestry systems (a land-use system that integrates trees and crops) is increasingly common around the world (Maia et al. 2021). Its adoption is particularly strong among smallholder farmers, while many countries consider it a vital strategy in implementing forest restoration activities (Stanturf et al. 2019; Mahmood and Zubair 2020; Stanturf 2021). Not only does it have the potential to restore degraded lands and overcome water scarcity, but it can also foster climate change mitigation and adaptation (Sharma et al. 2016; Favretto et al. 2018). It is proven to be a promising strategy for biodiversity conservation around the globe (Moreno-Calles et al. 2010; Sharma et al. 2016). It helps in increasing agricultural productivity and reducing rural poverty, thus addressing both the environmental and socio-economic objectives of rural development (Sharma et al. 2016; Brown et al. 2018). However, there are several limiting factors in the diffusion of agroforestry in developing countries (Sood and Mitchell 2009; Jara-Rojas et al. 2020), with Pakistan being no exception (Khan et al. 2017; Mahmood and Zubair 2020). Because of poor adoption strategies and high deforestation rates, rural communities are exposed to climate risks, including floods and droughts in the country (Mahmood et al. 2020; Ullah et al. 2021a).

Rural Pakistani communities are aware of the benefits of agroforestry adoption;, however, they face various constraints, including the expense of establishing trees, farmers’ poor access to credit, and little support from local authorities (Khan et al. 2017; Mahmood and Zubair 2020). Rural communities also have limited knowledge required for effective management of agroforestry systems and inadequate access to extension services (Khan et al. 2017; Dobson 2018). Additionally, a lack of access to capital and insecure land tenure contribute to these problems (Yasin et al. 2019). Thus, the poor diffusion of agroforestry is partially explained by a lack of fit between the technical aspects required for adoption versus the economic and institutional context of the different farming communities in which they are applied.

To make the diffusion of agroforestry systems successful for achieving the intended benefits of poverty reduction, it requires effective community-level participation in decision-making and the implementation of adoption and diffusion activities (Ullah et al. 2022). Local communities can provide effective methods for agroforestry diffusion and their management for the success of implemented strategies (Dlamini 2020). This is because community participation is inherent to the success of any project, and it increases social acceptance, as they are viewed as key beneficiaries and facilitators of implementation (van Os et al. 2016). Thus, social acceptance at the community level encourages diffusion of agroforestry systems, encourages people to manage the implemented measures, and establishes their trust, thus producing the desired outcomes in the form of changes in the environment and livelihoods with a positive behavioral attitude (Hughes et al. 2020).

**Fig. 1:**
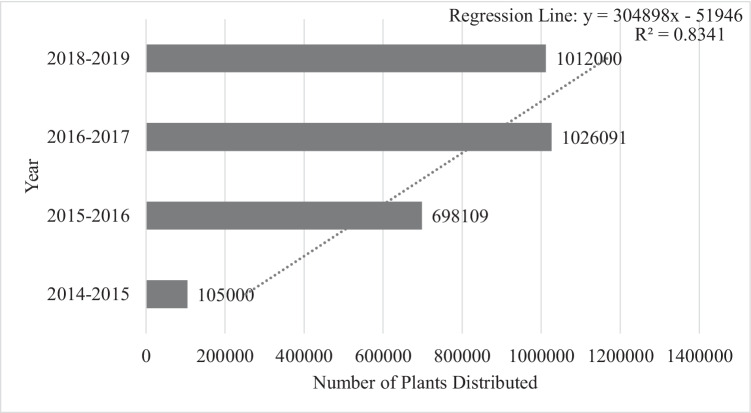
Distribution of plants under agroforestry

While a few studies examine the influencing factors of agroforestry adoption as an alternative land-use option using household level survey data in Pakistan (Khan et al. 2017; Mahmood and Zubair 2020), the adoption of agroforestry under BTAP in mountainous regions of KP is not well understood. To achieve the objectives of land restoration, climate risk mitigation, and livelihood improvements, the government of Khyber Pakhtunkhwa (KP) started a BTAP in 2014 (Ullah et al. 2021a). The BTAP sought to plan, design, and implement the “Green Growth Initiative” in the Forestry Sector of KP Province. The project was implemented by the KP government across the entire province, while the diffusion of agroforestry systems was a cover activity of this project, seeking to enhance human livelihoods by engaging the rural poor (Ullah et al. 2021a, 2021b). In the study region, under agroforestry/farm forestry willow, robinia, poplar, and ailanthus trees were widely distributed across farming communities. These plants were chosen without evaluating the interests of the larger farming communities and individual farmers. The plants were not appropriate for the context of many communities and farmers; thus, the willingness of farming communities and households in agroforestry adoption may have been harmed. Figure 1 shows the state of plants distributed under BTAP along with the trend line of plants distributed.

Therefore, we took the advantage to investigate the diffusion of agroforestry that had been in operation since the start of the BTAP project. The study aims to investigate those factors that restricted the FD in the successful diffusion of agroforestry and farming communities’ adoption of agroforestry under BTAP in KP, specifically taking the Dir Kohistan (Upper Dir) forest division as the study area. The findings will help to formulate guidelines for the successful diffusion of agroforestry under the new 10-BTAP.

## Methods

### Study area

**Fig. 2:**
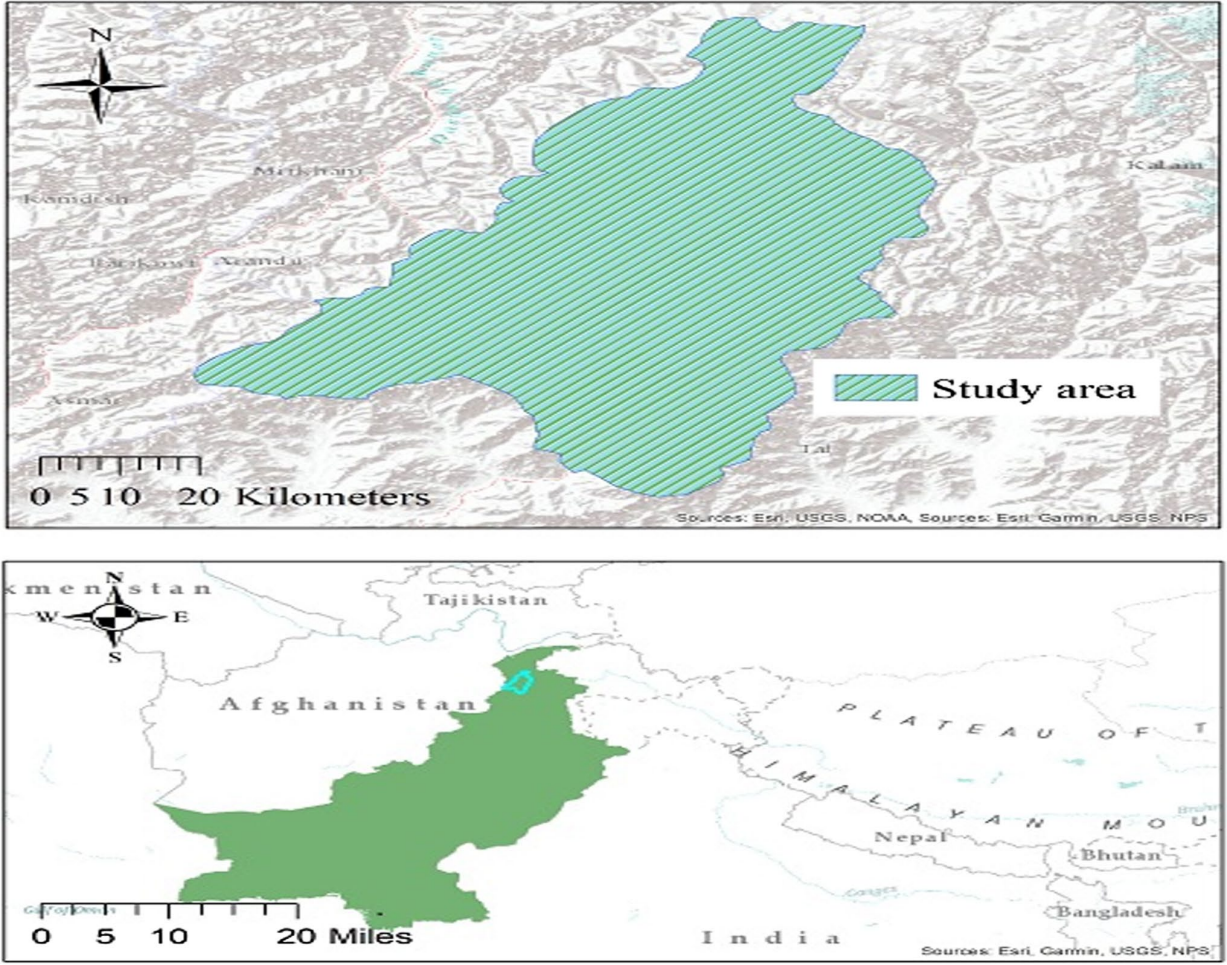
Map of the study area

The study was conducted in the Dir Kohistan forest region of the Upper Dir district (Fig. 2). The community resides in forest region III, Malakand forest region of Khyber Pakhtunkhwa (Ullah et al. 2021a). The region is located in the Hindu Kush Himalaya (HKH) Mountains and is characterized by frequent rainfall ranging from 1000 to 1600 mm a year and temperatures between 0 and 32 °C (Ullah et al. 2021b). This region is located at 35°9′ to 35°47′ latitude and 71°52′ to 72°22′ longitude and comprises 167,032.39 ha. Forest resources are the major livelihood activity in the region, including 56,822 ha of coniferous forest located at an altitude of 1677 to 5750 m (Ullah et al. 2021a, 2021b). Regarding cropping, rainfed smallholder farming is the major livelihood activity in the area. Major crops grown include maize, wheat, and potatoes. Because of deforestation, crop farming in the region is at a high risk of climate change and climatic extremes, viz., floods and droughts (Ullah et al. 2021a, 2021b). Crop fields are mostly located near Panjkora River and are exposed to frequent floods. Additionally, the community is endowed with forest resources that are economically and culturally very important for the area albeit with high deforestation and overgrazing of the forests. Since farmers lack alternative energy resources and also practice livestock production, large-scale raising of small ruminants continues in the area (Ullah et al. 2021b). The forest resources in the region are a rich source of timber products, non-timber products (e.g., honey, edible grasses, and seeds), fuelwood, medicine, tourism, and environmental services (Hussain et al. 2016). Since most farmers in the area depend on natural products extracted from the forest resources to sustain their livelihoods, the ongoing loss of woodlands and forest resources is a cause of concern for agricultural livelihoods.

Agroforestry was introduced in the study region as an important activity of BTAP in 2014 and was adopted by farmers rapidly (Ullah et al. 2021a). For generating cash income, firewood, and protecting crop fields, agroforestry was very popular among households in the study region. However, plants were distributed among farmers rapidly without any proper policy, planning, and assessment (Ullah et al. 2022).

### Data collection

In-depth interviews were used to collect qualitative data on the barriers and motivation of the diffusion of agroforestry at the community level under BTAP. In-depth interviews were conducted with VDCs members (*N* = 140), FD officials (*N* = 40), and community farmers (*N* = 300) in the study region. Under the project rules, free plants were distributed by FD employees among general farmers with VDC (community-based organization) involvement. The VDCs acted as a bridge between the community farmers and the FD in facilitating agroforestry diffusion. Under the rule, each VDC had 12 to 15 elderly farmers in membership, and each village had only one VDC (at village level). The in-depth interviews were first held with the VDC members in 10 different villages: Doog Dara (IDIV-01), Kal-Kot (IDIV-02), Thal (IDIV-03), Shaheed (IDIV-04), Cham (IDIV-05), Shokanay (IDIV-06), Dramdala (IDIV-07), Shahoor (IDIV-08), Barikot (IDIV-09), and Pattrak (IDIV-10). Then, in the same villages, in-depth interviews were again held but this time with farmers. Each member was contacted separately by the lead author. The VDC members of each village recommended potential adopters (general farmers) to the FD officials. All the 140 participants from VDCs were the elders of their tribes, and all were the key decision-makers at the community level. Similarly, out of a total of 62 employees, 40 FD officials responded to our in-depth interviews. All the 300 farmers were chosen for interviews randomly. Thus, the total of 480 respondents comprised VDC members, FD staff, and farmers (community members). A script was developed for in-depth interviews on barriers to the diffusion of agro-forestry programs initiated by the government FD under BTAP. The same script was used in each key informant interview. Each interview lasted between 1 and 2 h. All the interviewers were employees from the government FD, and each participant actively participated in the interview. Face-to-face interviews were the major data collection tool and were carried out from September 2020 through May 2021.

### Data analysis

#### Qualitative data analysis

The qualitative data analysis procedure is carried out through data transcription, a process of reproducing spoken words from in-depth interviews into written form (Winke 2017). We employed verbatim, a word-for-word translation, and transcription of recorded data (McNamara and Wood 2019). The transcription was done in order to identify key themes, similarities, differences, and respondents’ experiences (Hill et al. 2020). This process yielded data that was necessary for answering the research questions. Thereafter, we conducted content analysis to enable large volumes of data analysis efficiently by counting the number of times a phrase appeared in the transcription (Hagen 2018). This enabled us to determine the extent of a barrier and/or a motivator in agroforestry diffusion. The responses from participants and narratives were coded into themes that helped in making sense of shared meanings and experiences of the community. The thematic analysis was useful since all the data delivered a collective answer to all the barriers and motivators of farm forestry diffusion.

In the “Results and discussion,” comments from VDC members are represented as IDIV, whereas statements from general farmers are represented as IDIf. Statements of FD employees are quoted as IDIFD.

#### Quantitative data analysis

Since the focus of the study was farmer adoption of agroforestry under BTAP, the binary logit model was used to analyze those factors that affecting their adoption. As the decision to adopt agroforestry or not under BTAP was a binary decision, the analysis can be performed using binary choice models (Ullah et al. 2020a). Farmers’ decision is a dichotomous outcome (to adopt agroforestry or not to adopt) and is related to a set of explanatory socio-economic variables that are expected to influence the outcome (Mucheru-Muna et al. 2017). Therefore, a binary logit model was used to estimate the factors that affect farmer adoption of agroforestry under BTAP (Ullah et al. 2021a).

The logit model is expressed as follows:

The dependent variable is binary, which is the natural log of the odds (logit), that is;

$$\mathrm{logit}\left[p\right]=In\left[\mathrm{odds}\left(Y=1\right)\right]=In(\frac{p}{1}-p0)$$

$$\mathrm{logit}\left[p\right]= {\beta }_{0 + }{{\beta }_{1}X}_{1 + }{{\beta }_{2}X}_{2 + }{{\beta }_{3}X}_{3 + }\dots .. {{\beta }_{i}X}_{i +}{\varepsilon }_{i}$$

The dependent variable (*Y*) is the farmers’ adoption (0) or non-adoption (1) of agroforestry under BTAP, and *X* denotes a vector of the independent socio-economic and farm-related variables used in the study (Table 1). The Hosmer and Lemeshow goodness-of-fit tests for the binary logit model was used to determine whether our model is a good fit to the data; this indicated no significant difference between the predicted value and the observed value, showing a good fit of the model (with a *p*-value of 0.185 > 0.05).

**Table 1:** Description of the variables used in the binary logit model

Variables	Type of measurement	Expected signs	Adopters (S.D.)	Non-adopters (S.D.)	*t*-value
$y$— adoption of agroforestry	Dummy (1 if yes, 0 if no)				
${X}_{1}$— age	Numeric (years)	+	32.90 (10.13)	48.32 (12.71)	− 11.61***
${X}_{2}$— education	Numeric (years)	+	3.72 (4.91)	3.64 (4.67)	.157
${X}_{3}$— farm size	Numeric (acre)	+	4.67 (3.38)	1.33 (0.93)	11.66***
${X}_{4}$— family size	Numeric (years)	+	14.88 (5.59)	10.90 (2.20)	8.10***
${X}_{5}$— dependence on firewood for cooking and heating	Dummy (1 if yes, 0 if no)	+	0.90 (0.31)	0.10 (0.30)	− 22.001***
${X}_{6}$— access to information	Dummy (1 if yes, 0 if no)	+	0.86 (0.48)	0.14 (0.30)	− 11.40***
${X}_{7}$— forest cover	Dummy (1 if yes, 0 if no)	−	0.12 (0.30)	0.88 (0.43)	15.18***
${X}_{8}$— crop cover	Dummy (1 if yes, 0 if no)	+	0.88 (0.49)	0.12 (0.26)	− 10.43***
${X}_{9}$— negligence of a farmer by a forest employee	Dummy (1 if yes, 0 if no)	−	0.01 (0.01)	0.99 (0.42)	21.72***
${X}_{10}$— ownership of animals	Dummy (1 if yes, 0 if no)	+	0.57 (0.44)	0.43 (0.49)	− 3.28***

^***^ denotes statistical significance at the 1% level

### Variables used in the logit model

Table 1 presents the definition and measurement of independent variables included in the logit analysis. Previous studies show that farmer (household head) socio-economic and farm-related characteristics affect the adoption of agroforestry (Beyene dafet al. 2019; Ullah et al. 2021b). The household head’s age positively affects farmer adoption of agroforestry practices (Beyene et al. 2019). This means that the likelihood of a farmer to adopt agroforestry is greater among old-aged farmers than young farmers. The probable reason for adoption among older farmers could be that, in traditional societies, such farmers have control over their households and, due to their farming experience, they can perceive the benefits of agroforestry adoption. In studies on household adoption of agroforestry, education is an important variable that positively influences farmers’ adoption decisions (Lasco et al. 2016; Ullah et al. 2021b). This might be because the educated farmers have more knowledge and understanding of the benefits of new practice adoptions in agriculture. In studies of agroforestry adoption, farm size is proven to be an important variable that positively influences farmers’ adoption decisions (Dhakal et al. 2015; Beyene et al. 2019). The positive relationships of farm size with adoption might be because the size of a farm land owned indicates household wealth status in the study region. Family size is also an important variable that positively affects household adoption of agroforestry practices because agroforestry is labor intensive (Sabastian et al. 2014; Dhakal et al. 2015; Beyene et al. 2019). Therefore, we expect a positive relationship between farmer family size and the probability of his/her adoption of agroforestry practices. Previous studies find that a farmer households’ high dependency on fuelwood for cooking and heating positively affects his/her adoption of agroforestry practices (Toth et al. 2019; Singh et al. 2021). Therefore, a household’s high dependency on fuelwood for cooking and heating is expected to be positively associated with the likelihood of a farmer adoption of agroforestry. Access to information helps farmers choose strategies that enable them to cope with fodder and firewood scarcity through the adoption of agroforestry (Ullah et al. 2021b). Therefore, we expect that access to information will positively affect farmers’ adoption of agroforestry. Ullah et al. (2021a) report low adoption of agroforestry among farmers in those communities where the land area is primarily covered with forest. Thus, we expect that the forest cover of a village may negatively affect farmers’ adoption of agroforestry. Alternatively to forest cover, increased crop cover (farmers in those communities where the land area is primarily cropland with little or no forest areas) may positively affect a farmer’s adoption of agroforestry. Crop cover is expected to be positively associated with the likelihood of a farmer to adopt of agroforestry. Ullah et al. (2021a) report that a good relationship of FD staff with farmers is a motivator in adoption of forest restoration initiatives. We expect that negligence of a farmer by a FD staff will negatively affect their adoption of agroforestry. The ownership of animals affects the household decision to adopt agroforestry (Ullah et al. 2021b). Thus, ownership of animals or livestock is also included in the study, positively correlated with agroforestry adoption.

## Results and discussion

### Descriptive statistics

Table 1 also shows summary statistics of the independent variables used in the logit model. The results in Table 1 suggest significant differences between agroforestry adopters and non-adopter farmers under BTAP in terms of their socio-economic and farm-level features. Concerning farmers’ demographic characteristics, such as age, farm size and family size, our results indicate that adopters of agroforestry have significantly higher average farm and family sizes but are younger than their non-adopting counterparts (Table 1). Furthermore, dependency on firewood for cooking and heating was significantly higher among adopters of agroforestry. The results show that of all respondents dependent on firewood collection for cooking and heating, 90% adopted agroforestry (Table 1). In addition, agroforestry adopters had significantly more access to information than non-adopters. Of all farmers who showed access to information, 84% adopted agroforestry (Table 1). We also found significant differences in the village forest cover of adopters and non-adopters. Our results show that 88% of the non-adopter had greater forest cover in their villages than adopting farmers (Table 1). Similarly, 88% of the adopting farmers had greater crop cover in their villages than non-adopters. There were significant differences between adopters and non-adopters with respect to crop cover in their villages. Our results further show that about 99% of the non-adopters report that FD employees ignored them with respect to the selection of plant species and/or other decisions regarding agroforestry adoption (Table 1). It is also reported that most adopters of agroforestry possess animals, especially small ruminants. There is a statistically significant difference between adopters of agroforestry and the non-adopters in terms of livestock ownership (Table 1).

### Significance of agroforestry diffusion in HKH

**Fig. 3:**
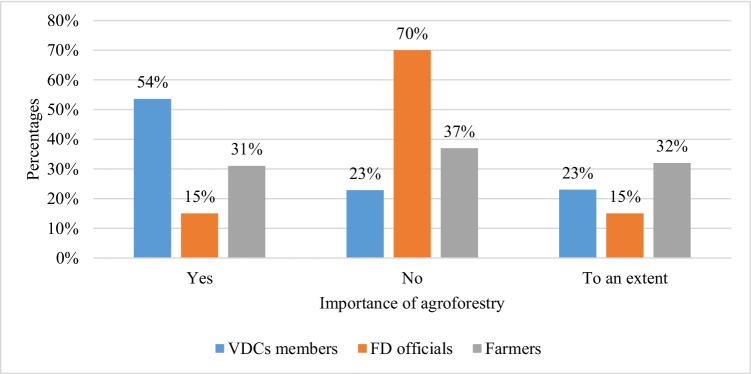
Community elders, farmers, and FD officials’ views of agroforestry importance in the HKH

The results of in-depth interviews show that a majority of VDC members and a huge majority of farmers understand the importance of agroforestry diffusion in the study area (Fig. 3). It is also observed that those farmers who showed importance of agroforestry in the study area usually had riverside crop lands, and their communities were involved in agricultural activities. We also observe that those communities involved in crop farming and had agricultural lands on the uphill showed the significance of agroforestry diffusion to an extent (Fig. 3). Moreover, communities residing on the forest margins and poorly involved in crop farming show no significant agroforestry diffusion (Fig. 3).

Surprisingly, the results of in-depth interviews from the FD officials reveal completely opposite views, where most respondents show no significance of agroforestry in the study area (Fig. 3). There could be two reasons for the completely opposite results of in-depth interviews from the FD employees from those of community elders. The first is that throughout history the forest employees have performed their duties in forest areas and were barely trained for delivering services to crop farmers. This was because many forest employees had the prejudiced view that these communities are forest dependent and do not need trees for crop fields. An FD employee reported that: “What will be the importance of eucalyptus or willow in a community residing in a deodar forest?” (IDIFD-03). The other reason observed was extra duties given to FD employees during the project period. An FD employee reported: “I have not visited home for a month. Yesterday I decided to go home and when I reached near to my home, the laborers from the nursery called me to say that many crop farmers had come to the nursery to collect plants. I returned without reaching home to my work place” (IDIFD-33).

### Respondents’ perceptions of agroforestry adoption motivating factors

#### Crop lands’ location

Our results show a clear similarity in perceptions of FD officials, farmers (adopters), and VDC members, where majority of the respondents (87.5, 87.3, and 86.43%, respectively) from all 3 groups agree that the location of farmer’s fields (Table 2) affect the adoption of agroforestry. The respondents in all three groups clearly perceive that those farmers with agricultural lands on riversides usually adopted agroforestry in the BTAP program (Table 2). The majority of respondents in all 3 groups believe that farmers with riverside fields expect that they can increase farm yield by adopting agroforestry.

**Table 2:** Motivating factors of agroforestry adoption

Variables	FD officials		Farmers		VDC members	
	Cases	Percentage	Cases	Percentage	Cases	Percentage
Crop land location	35	87.5	131	87.33	121	86.43
Provision of firewood	29	72.5	111	74.00	97	69.29
Timber value of agroforestry	21	52.5	77	51.33	83	59.29

In the farmers’ column, the table shows the perceptions of agroforestry adopters only

According to FD officials, farmers (adopters), and VDC members, agroforestry adoption was a necessity of local communities, since the majority of households’ crop lands were located along the riverbank. Communities facing dozens of problems know that by adopting agroforestry they can mitigate many of these problems. A VDC member from a household whose crop fields were riverside reports: “All our cultivated land is on the riverbed. Since our ancestors, we have been doing agriculture on the riverbed, and near the riverbank. Every year in the rainy season we are facing floods that make our agricultural production very risky. Adopting agroforestry can help us to mitigate several risks and disasters and therefore, farmers are quick to adopt agroforestry in our community” (IDIV-04). Another elderly farmer reports, “ … When flood occurs, then we lose all crops, which means our entire labor, time, and money are wasted. Not only our crops but also our livestock die. We know the place is dangerous, but our earnings from farming upland are not enough to meet our needs. If the FD is helping us in providing skills and free plants then we will adopt agroforestry since this adoption is our need. It will also resist floods” (IDIf-01)*.* Similarly, another community elder reports, “ … Agroforestry can contribute to the mitigation of climate change while delivering multiple benefits to farmers. Farmers are exposed to climate variability, land degradation, and poverty in the area whereas; agroforestry adoption can help farmers to cope with these challenges” (IDIV-03). Another elderly man from a household whose land is along the river responds: “My land is on the river side. Agroforestry adoption is my need” (IDIV-03).

These results indicate that to support the diffusion of agroforestry in a larger community, national governments, especially when using finance to protect local communities from climate risks, need to focus more on the planning of adoption activities of farm forestry than on the free distribution of trees in the face of climate extremes, which are similar to the findings of Mahmood and Zubair (2020). Our findings also confirm the results of Quandt (2020) by providing evidence that, in developing countries, many farmers have realized the importance of agroforestry adoption in mitigating climate risks; however, governments are also anticipatory adoption actors and need to play their roles.

Thus, a main factor shaping the motivation of farmers to adopt agroforestry is land location. The policy to promote agroforestry is to increase the diffusion of agroforestry in those communities where farmers have lands along rivers. Ollinaho and Kröger (2021) also show support for this policy as they report massive diffusion of agroforestry across similar geographical areas. In communities where crop lands are not along rivers, further information may be disseminated on agroforestry/farm forestry, free distribution, plant availability, and the benefits of adoption to such communities. Before agroforestry diffusion in communities where land is not on riverside or they reside on forest margins, dissemination of information and large scale awareness will enhance agroforestry adoption. It is expected that farmers’ involvement in agroforestry on riversides and dissemination of information on agroforestry and BTAP to farmers residing in other locations will benefit both communities, and the adoption will be motivated among those farmers as well who do not have crop lands on river sides, or either they have crop lands on forest margin. Ollinaho and Kröger (2021) suggest similar policies, including providing information on agroforestry as a policy intervention that promotes agroforestry diffusion and establishes secure land in degraded lands or areas with challenging environments.

#### Dependency on firewood collection

FD officials, farmers (adopters), and VDC members perceive that community dependence on firewood, that is the role of agroforestry in providing firewood, is the second most important factor motivating agroforestry adoption (Table 2). About 72.5% of FD respondents, 74.0% of farming community respondents, and 69.29% of VDC members believe that adopters implemented agroforestry because they and their community need firewood for cooking and heating (Table 2).

The FD and VDC respondents specifically reveal that the community members have realized that agroforestry adoption can control deforestation while also meeting fuelwood needs and addressing fodder scarcity. The area lacks alternative sources of energy, with both heating and cooking highly dependent on firewood and forests. Farmers also report that the community realized that the agroforestry adoption can protect key forests from degradation and can provide a pitch for sustainable harvesting of trees to meet household firewood needs and, therefore, they adopt agroforestry. An FD official and a farmer report, “There is an increasing recognition of the potential role of agroforestry adoption in the provision of sustainable firewood in the region which has motivated agroforestry adoption to a large extent” (FD-29 and IDIf06). Similarly, a VDC member reports, “Households in my community always used to damage forests to meet their firewood needs. Because there were no other options with them to meet their energy needs. When the government started free distribution of plants, I acknowledged the protective and productive values of agroforest adoption and directed my community to collect free plants from the FD” (IDIV-01).

This is similar to the findings of Faße and Grote (2013) and Sharma et al. (2016) who report that households that do not adopt agroforestry typically have access to alternative sources of heating and cooking. Faße and Grote (2013) report that poor households with greater dependence on natural resources usually have consciousness about the adoption of agroforestry, since it is their only source of firewood.

The policy implication is that, due to frequent harvesting of pine forest, agroforestry adoption has a good potential. Community wood dependency is found to be a major factor enhancing agroforestry adoption. Therefore, agroforestry should be disseminated more in communities that are dependent on firewood. However, performance of FD in those communities that have access to other sources of energy should be increased for the intended success of the current agroforestry program under BTAP. This may include restructuring the public sector and community-based institutes that are involved in the dissemination of agroforestry with the aim of improving the quality of service provision through restructuring these organizations. Similar policy is reported by Biland et al. (2021) as they suggest policies for improving FD performance with respect to the diffusion of forest trees on farms.

#### Timber value of agroforestry

Finally, regarding FD officials, VDC members and farmer perceptions of the potential motivators of agroforestry adoption, the results show that the timber value of agroforestry is also an important factor motivating agroforestry adoption under BTAP (Table 2). The timber value of agroforestry is perceived as important for adopting agroforestry by about 52.5% of the FD officials, 51.33% of the VDC members, and 59.29% of the farmers (Table 2).

The FD and VDC members reveal that trees from agroforestry have a great market value in the region. Since trees are a major source of income for farmers, adopting agroforestry is a good motivator leading to timber harvesting. A member of a VDC reports, “Because of the essential role of agroforestry for providing timber, the adoption was quick among households” (IDIV-07). Similarly, an elderly farmer who was a member of a VDC reports, “Agroforestry was adopted in my community due to their high timber value and huge use as a forage for livestock” (IDIV-09 and IDI-05). The FD official also confirms this, relating to the quick diffusion of agroforestry among communities because of its higher timber value. An FD official reports*,* “Because of their higher timber value and market price, many farmers, especially smallholders were quick to adopt agroforestry under BTAP” (IDIFD-21).

This result is similar to the findings of Sabastian et al. (2014), who find that the timber value of agroforestry determines the participation of farm households in agroforestry programs and practices. The policy implication for farmers adopting agroforestry is disseminating information on timber and economic values of agroforestry in communities which are not aware of the much-needed timber value of agroforestry adoption and is suggested by researchers (Biland et al. 2021; Ullah et al. 2022). Policy promoting agroforestry should increase the diffusion of agroforestry in communities where farmers have small landholding sizes, since they are more in need of and responsive to, agroforestry adoption. In such communities, agroforestry will increase crop productivity and will provide income to small landholders. Big farmers with large landholdings should also be convinced and motivated to adopt agroforestry. It is expected that small and big farmers will have equal involvement in agroforestry under BTAP, experiencing equal benefits.

### Factors influencing household level adoption of agroforestry under BTAP

Results of our analysis show that the households’ head age has a significant (*P* < 0.01) and positive influence on the farmer decision to adopt agroforestry under BTAP (Table 3). This means that an older farmer is more likely to adopt agroforestry than a younger farmer. Previous studies report that older farmers adopt agroforestry as they have more rights and resources available, thus signifying their better position to adopt agroforestry (Gebru et al. 2019; Jha et al. 2021). Jha et al. (2021) further reports that younger farmers may find the benefits and market responses from agroforestry to be too slow; thus, they are expected to be slow to adopt versus older farmers. This finding is in line with previous studies: both Coulibaly et al. (2017) and Beyene et al. (2019) show that older farmers are more likely to adopt agroforestry than younger farmers.

**Table 3:** Results of the logit model

Variables	Coefficient	Standard error	Wald *χ*^2^	Sig	Odds ratio
Age	0.188	0.078	5.831	0.016	1.207
Education	− 0.100	0.123	0.669	0.413	0.905
Farm size	− 0.465	0.432	1.162	0.281	0.628
Family size	− 0.127	0.158	0.644	0.422	0.881
Dependence on firewood for cooking and heating	1.363	1.481	0.847	0.357	3.908
Access to information	3.607	1.528	5.575	0.018	36.869
Forest cover	− 4.587	1.509	9.247	0.002	0.010
Crop cover	4.792	2.332	4.222	0.040	120.518
Negligence of a farmer by a forest employee	− 21.524	2923.671	0.000	0.994	0.000
Ownership of animals	2.316	1.442	2.577	0.108	10.130

Summary statistics: − 2 log-likelihood = 24.734; pseudo-*R*^2^ = 0.729; Prob > *χ*^2^: 0.00

The probability of adopting agroforestry is significantly (*P* < 0.01) and positively affected by farmer access to information (Table 3). This means that a farmer with better access to information on the benefits of agroforestry adoption, the procedure through which a farmers can participate in BTAP for adopting agroforestry, and the species available under agroforestry free distribution program of the project were more likely the adopters of agroforestry as compared to the farmers with low access to information. Thus, our study identifies that farmer access to information is an important variable shaping the adoption of agroforestry under BTAP. Our results are in line with the findings of Martini et al. (2017) and Buyinza et al (2020), both reporting that access to accurate information is very important for farmer adoption of agroforestry practices. They further report that the lack of access to information is often related to farmers’ poor social connectedness which complicates farmer behavior toward the adoption of agroforestry.

There is a usual lack of the evidence on the effect of increased forest cover (as compared to crop cover) in a village on community and household adoption of agroforestry practices. Confirmed through the in-depth interviews and personal observations through staying for a longer time inside the community, we observe that increased forest cover (as compared to crop cover) lowers farmers’ enthusiasm to adopt agroforestry. This is because farmers in such communities had dense pine forests through which they were already receiving higher incomes. Furthermore, they view that the pine trees are easy to burn, fulfilling their firewood needs (wood can be easily obtained from these forests). Therefore, they are not interested in adopting agroforestry under BTAP. This view is confirmed by our analysis, with significant results (*P* < 0.01), showing a negative relationship between forest cover and farmer adoption of agroforestry (Table 3).

Similarly, as expected, increased crop cover in a village shows a positive and significant (*P* < 0.05) relationship with the probability of a farmer to adopt agroforestry under BTAP (Table 3). This means that respondents with less forest cover and more crop cover are more likely to adopt agroforestry. Personal observations and key informant interviews with community members with more crop cover show that farmers in such communities adopt agroforestry to diversify their income and fulfill their firewood needs. The other reason behind adoption of agroforestry under BTAP in such communities is this that most croplands in such communities are situated along rivers, thus adopting agroforestry helps protect crops from wind (windbreak) and also prevent soil erosion. The high adoption among such communities might be because they have large crop lands. This result is consistent with the findings of Beyene et al. (2019), with their results suggesting that the more a farmer is involved in agricultural crops, the more they adopt agroforestry as alley cropping to improve crop production.

Household-level attributes play an important role in agroforestry adoption. However, there are external factors, like the lack of trained staff, lack of budget, a communication gap between forest staff and communities, poor species selection, and limited community interest that limits the adoption of agroforestry practices.

The policy implication is to motivate farmers who reside in villages, where forest cover is greater and communities are near forest margins because a main factor undermining the motivation of farmers to adopt agroforestry is the forest cover in a village. Thus, the policy is to increase awareness in such communities on the importance of forest restoration and the role of agroforestry adoption in forest restoration. The other important policy motivating agroforestry adoption is to use older farmers. This is because the older farmers are considered village elders, thus key decision-makers whose instruction or agreement on agroforestry adoption can also motivate young farmers. Information provision is an important policy that can create awareness and interest among farming households. Access to information, especially for new adopters, can motivate many non-adopters in mountainous and remote regions with very limited resources to adopt agroforestry. It is expected that involving farmers in those villages where crop cover is greater than forest cover will enhance agroforestry adoption. These households will benefit through greater incomes and increased confidence in agroforestry. Such policies are also suggested by researchers in other parts of the world (Ota et al. 2020; Shennan-Farpón et al. 2022).

### Factors influencing the diffusion of agroforestry by Forest Department

#### Farmers’ disagreement in front of monitoring teams

Our results show that all (100%) FD officials and 55.71% VDC members participating in our interviews reveal that many farmers have refused plants or any other services from the forest department when approached by monitoring teams (Table 4). Denial of plants and services discourages FD officials from disseminating agroforestry in the region. The respondents from FD and VDCs perceive that previously FD employees used to provide free plants to every farmer; however, now they carefully decide about who gets plants and who does not. FD employees are reluctant to provide farmers free plants if previously given plants under agroforestry/farm forestry free distribution and are not acknowledged in front of monitoring teams (Table 4).

**Table 4:** FD and VDC member perceptions of barriers to agroforestry diffusion

Variables	FD officials		VDC members	
	Cases	Percentage	Cases	Percentage
Farmers'’ denial of services in front of monitoring teams	40	100	78	55.71
Non-availability of extension staff	37	92.5	73	52.14
Misunderstanding/miscommunication among project staff and community	33	82.5	70	50.0
Lack of sufficient budget for activities	40	100	61	43.57

Many FD officials reveal that they provided free plants to farmers to grow in their crop fields. Then many farmers, if not all, denied having collected these free plants to our officer and monitoring teams. An FD official reports, “Throughout the years, everyday I convinced dozens of crop farmers to adopt agroforestry and provided them with hundreds and even thousands of free plants. And then in front of the monitoring team at the end, I was surprised when I heard from them that they have not collected any free plants” (IDIFD-17). The elderly farmers of the community also agree with the statements of FD officials, reporting that many community members who denied having received free plants in front of the monitoring team had, in fact, received free plants. A VDC member reports, “When they disagreed in front of the monitoring team, I asked them why they were disagreeing? Tell the monitoring team that you have received plants… And then…in front of the monitoring team many farmers used to say in my ear that none of the plants has grown. If I agreed that I have collected free plants but all the plants have failed then the team may not take money from me” (IDIV-08). Both the FD officials and the community elders (VDC members) agreed that this frequent refusal made the FD department ashamed, and next year they were not willing to give free plants to every farmer. An FD official reports, “Why I work so hard for the diffusion of agroforestry in such a tough hilly region when it will bring shame for me in the end” (IDIFD-13). Similarly, another FD official reports, “Why do I work day and night? Just to disgrace me?” (IDIFD-39). The VDC members and FD officials report that, at the start of BTAP, the FD officials were working hard to diffuse agroforestry across the region. They were seen visiting farms and homes, convincing masses to adopt agroforestry. They provided free plants to farmers at their doorsteps. However, after monitoring team visits they were worried, and no longer visiting farms and homes. Even if farmers visit their office or nursery, the FD officials are still reluctant to provide free plants. An FD official reports, “Now I do not give free plants to any farmer until I know him personaly” (IDIFD-09). Another FD official reports, “I only provide plants to those farmers whom I am confident that tomorrow if someone asked them that they have collected free plants and they would tell him that of course…yes” (IDIFD-11).

This is similar to the findings of Creasy and Anantatmula (2013): they find that good monitoring reports increase staff satisfaction and their willingness to contribute further to project goals. In similar situations, Chaudhuri et al. (2021) suggested a policy to convince farmers to become connected with community members and take relevant recommendations from them on different activities for sustainable development. Thus, the policy recommendation is to convince farmers to acknowledge the goods and services they receive from the FD by developing connections with and among community members. It is important that VDC members and community elders run a campaign to make farmers understand the value behind acknowledging plants in front of monitoring teams. Furthermore, monitoring teams should make frequent visits to farms, and make a specified schedule of visits so that the FD can work efficiently. The monitoring should be fair, including community elders, and VDC members. It is important that the VDC members and community elders work with the FD and to make the monitoring of agroforestry fair and smooth. The monitoring teams also ensure and allow the forest department to disseminate information among farmers without fears.

#### Non-availability of extension staff

Non-availability of extension staff in the forest department is also considered an obstacle by 92.5% of the responding forest department officials and 52.14% of the responding VDC members; this hinders the diffusion of agroforestry in the study region (Table 4). The respondents reveal that access to extension services is important in remote regions where low literacy hinders adoption of productivity enhancing agricultural practices. However, the respondents reveal that extension services are not available to local communities, since the forest department does not have any extension agents who might effectively disseminate agroforestry across farming communities (Table 4).

In particular, FD officials report that throughout the BTAP, there was no staff of extension service providers. Without frequent extension contact, the diffusion of agroforestry is difficult. The village elders in Dir Kohistan said that they were not aware of the BTAP for a long time; then after becoming aware, they were unwilling to participate because they perceived that they had insufficient technical knowledge to adopt agroforestry practices and did not want to invest time and energy in “worthless activity*.*” An elderly man reports, “The extension service providers had no regular contact with community members and therefore they did not provide updated information about the project or information regarding agroforestry*”* (IDIV-06). Similarly, another elderly farmer reports, “Throughout the project, we did not see the extension agent. While running such a big project, the government should have ensured that all farmers have frequent extension contact” (IDIV-03). Moreover, the community elders report, “Because of the poor extension contact the farmers in the area awaited to see before they adopted” (IDIV-04). The FD official reported that there were no extension agents in the entire area who might have facilitated the diffusion of agroforestry in the valley. An FD official reports, “There was no extension staff with us. However, after extension agent transfer to the project area, a frequent extension contact was established among community members and project staff. The frequent contact cleared the community concerns about the BTAP and agroforestry diffusion.” (IDIFD-11).

Studies reported that, in Pakistan, the extension-farmers’ contact is very low, thus affecting adoption of new agriculture and forestry practices by farmers (Ullah et al. 2021b). Weak links between extension workers and farmers are often observed in the developing world (Ullah et al. 2020b, c). Therefore, predictions of agroforestry adoption need to account for the frequency and effectiveness of extension-farmer contacts. The policy implication is that the government should increase the number of extension agents across regional forest divisions. Such policy is also supported by Ullah et al. (2020c), as they suggest that the provision of extension services can help farmers and improve agricultural practices. Thus, extension workers should be placed in each forest division. There should be a separate staff working under each extension officer. The primary role of the staff should be to facilitate the provision of extension services. The availability of extension staff will not only facilitate awareness of agroforestry but also ensure the diffusion of other novel practices.

#### Misunderstanding/miscommunication among project staff and community

Apparently, the lack of extension services has resulted in miscommunication and misunderstanding among FD officials and community members (Table 4). About 82.5% of the FD officials and 50.0% of the VDC members responding to our interviews reveal that misunderstanding and miscommunication among project staff and community members hindered the diffusion of agroforestry in the study region (Table 4).

In-depth interviews with both VDC members and FD officials show that there was confusion among community members, making it difficult for the FD to disseminate agroforestry. An FD official reports, “The community were afraid of adopting agroforestry. They were afraid that their adoption would not yield. They were afraid that the agroforestry may not succeed on their farms. They were afraid that if agroforestry failed at their farms then the FD or project staff may not ask for account” (IDIFD-34). The community elders (VDC members) also agree with the FD officials’ statement, revealing that the fear in the community members’ minds was mainly due to the poor understanding of BTAP. A VDC member reports, “Many farmers did not adopted agroforestry. They were afraid that the government may not capture their lands by providing free plants and helping them in plantation activities” (IDIV-03). The in-depth interviews in all villages shed more light on the issue, where the majority of respondents claim that no project staff or FD officials informed them clearly about what they want to achieve by providing free plants. An elderly farmer (VDC member) reports, “They only keep saying, we want to promote plants which you can use as firewood in future. Sometimes they said, we want to improve your condition against floods and drought. But, many community members were not sure about what they mean by providing plants for free. Many believed that the government wants to capture their land through planting plants on the fields” (IDIV-02). A farmer who was well respected in his community reports, “How a community will adopt agroforestry when the government wants to promote the planting of billion plants but the community members, including the elders, thought it is going to capture community lands” (IDIV-01). Similarly, an elderly farmer reports, “There was no clear understanding of government objectives she wanted to achieve through this activity in the farming community's minds. After a year or two, a team came from Peshawar (the capital city of this province). They made them understand what the objectives are and the purpose of the agroforestry free distribution. Even now many have confusion in their minds and if it goes like that, miscommunication among the community will result in low diffusion” (IDIV-04)*.* Similarly, an another VDC member claims, “You should tell your staff (FD officials) to make it clear to the community that what they aimed through this activity in detail — like first objective, second and third, yes that is how it would increase adoption among the larger community” (IDIV-05).

This is like the findings of Zikargae (2018), who finds that misunderstanding, miscommunication, and conflicting issues between the community and the government creates a disharmony between sustainable development and environmental protection activities. Since a main factor undermining the motivation of farmers to adopt agroforestry is a miscommunication and a misunderstanding among stakeholders, especially the FD and potential adopters, the policy implication is to convey messages clearly to all farmers. This policy is also supported by Zikargae (2018), who suggests that effective communication can reduce misunderstanding and ensure sustainable development. In this connection, the FD should use multiple sources and media. Use of multiple sources for informing communities on the aims of agroforestry diffusion and BTAP will enhance agroforestry diffusion. It is important for the FD to ensure that the farming community understands the objectives and aims behind agroforestry. It must not only be ensured that the farmers have understood the message but also that the farmers have understood the message correctly. The involvement of farmers in agroforestry activities by FD will help them to build farmers’ confidence and make them understand the activities and the reasons behind different activities the government is conducting in the BTAP.

#### Lack of sufficient budget for activities

Non-availability of sufficient funds and provision of funds in time also limit agroforestry diffusion activities (Table 4). All the FD officials and 43.57% of VDC members responding to our interviews reveal that non-availability of sufficient funds and provision of funds in time hindered the diffusion of agroforestry in the study region (Table 4). In-depth interviews with FD officials show that there was a lack of funding for nursery raising, which also restricted agroforestry diffusion in the study area. An FD official reports, “There were only 15,000 Pakistani rupees (94 US dollar) for leasing private land for establishing a nursery. Therefore, in the entire valley, no one was willing to lease us land for establishing a nursery” (IDIFD-05). Similarly, another FD employee remarks, “No one was willing to provide us land on lease for establishing a nursery. The farmers used to say, “Why do I provide you land on 15,000 Pakistani rupees? And why do I not cultivate my land with maiz and save 100,000 Pakistani rupees instead of 15,000?” (IDIFD-27).

Since a main factor undermining FD efficiency in diffusing agroforestry in full across farming communities is the lack of funding; therefore, the policy implication is that the government should ensure the timely flow of funds to all divisions so that the FD can execute all of its missions. If the FD has funding, it can purchase plants and pay salaries to laborers and nursery owners in a timely fashion, thus accelerating agroforestry diffusion across farming communities. Furthermore, Molin et al. (2018) suggest that the FD may minimize costs on activities that are of low importance and adopt landscape approaches that reduce restoration costs.

### Factors influencing the diffusion of agroforestry by VDCs in farming communities

#### No participation of VDCs in planning and monitoring of agroforestry program

Participatory approaches to landscape restoration projects enhance agroforestry diffusion; however, our results reveal that some key stakeholders were not involved in the planning of agroforestry diffusion (Table 5). About 90.0% of the responding farmers and 87.86% of the VDC members report that the lack of participation by VDCs in planning and monitoring of agroforestry is an important factor that hindered agroforestry adoption by farming households and communities (Table 5).

**Table 5:** Farmers and VDCs member perceptions of barriers to agroforestry diffusion

Variables	Farmers		VDC members	
	Cases	Percentage	Cases	Percentage
No participation of VDCs in planning and monitoring of agroforestry program	270	90.0	123	87.86
Lack of needs assessment	244	81.33	98	70.00
Poor quality of distributed plants	223	74.33	81	57.86
Poor know-how of effective plantations	216	72.00	76	54.29
FD should keep promises so that the community can trust them	201	67.00	72	51.43
Wastage of plants	153	51.00	61	43.57

Participation of local communities in agroforestry planning and implementation is considered critical for executing agroforestry and afforestation activities sustainably. The participants in in-depth interviews argue that increasing local people’s involvement in agroforestry and afforestation will make it easier for the government to achieve its objectives sustainably. An elderly farmer reports, *“*To be able to make agroforestry successful and increase the crop productivity and income, the Plan should be made in community consultations and should be implemented accordingly” (IDIf-06). Another elderly farmer who is well respected in the community reports, *“*The government needs to ensure through proper monitoring that the plan is made through community participation and implemented accordingly and successfully” (IDIV-07). Community elders suggest that the project will only achieve its objectives successfully if the community participates in decision-making. An elderly man reports, *“*If the benefits will be for everyone including poor and small farmers then the outcomes of the project will be tangible. However, visible results encourage that plans are made through community consensus” (IDIV-05).

Many in-depth interview respondents report that they want to establish good relationships with anyone who is helping them technically and who is providing free plants. The VDC members state that at one location, the FD asked the VDC of village elders to recommend potential adopters; however, on the other hand, they never invited them to make plans for diffusion of agroforestry. Their role was only to sign on a farm in the village, recommending him to the FD for the free plants. The FD never asked VDC members about which plants were best suited or how to disseminate them effectively. VDC participation in project planning and monitoring can make agroforestry diffusion successful. An elderly farmer who was a key decision maker of his community reports, “Not the government…not the people from the FD…not the people from the agriculture department…instead, we consider them our brothers that are helping us. These are the people that want to work with us and for us. And if people, who are promoting agroforestry in our areas want to work for us then, they need to work with us and get us involved in planning activities for promoting agroforestry” (IDIV-07).

The communication gap between government actors promoting agroforestry and farming communities makes divisions of responsibilities unclear in adoption processes (Selman 2004; Khoshkar et al. 2020). These results are similar to those of Mengistu and Assefa, (2020). They report that the sustainability of environmental management programs depend on the extent of community participation in planning and implementation. Zero-community level participation negatively affects adoption. Thus, our results show that the absence of VDCs from planning and implementation of agroforestry reduces the adoption of agroforestry by community members. It also reduces the capability of VDC members to effectively disseminate agroforestry in their communities. Therefore, policy should require the involvement of VDCs in planning and implementation of agroforestry/farm forestry programs; as also recommended by Mengistu and Assefa (2020). As VDCs need to be proactive in diffusing agroforestry across their communities, their involvement in agroforestry activities and initiatives is critical. Similarly, the VDCs, through involvement in decision making, can link community farmers directly with the FD, thus improving agroforestry diffusion.

#### Lack of needs assessment

Our results show that 81.33% of the farmers and 70.0 VDC members reveal that plants were distributed without consideration of the farmers'’ needs (Table 5). They further reveal that this lack of assessment limited diffusion of agroforestry in the region (Table 5). The respondents reveal that the plant needs assessment is vital for identifying which plants are more liked or preferred in the region and that the dissemination of desired plants will make farmers quickly adopt agroforestry. They identify that the needs assessments would have helped to identify plants and species needs of farmers; however, this lack of needs assessments meant that the distributed plants were of little or no interest to the farmers and, thus, can be attributed to the non-adoption of agroforestry by many communities.

Despite the availability of free plants for distribution under agroforestry, their available plants were not what farmers wanted. In in-depth interviews, most farmers report that the availability of free plants was not a significant factor in motivating farmers to adopt agroforestry, but it was the plants of farmers’ interest (Table 5). An elderly farmer said, “The free distribution of plants was a motivator to a very small extent since the FD was providing plants in which the farmers did not have any interest” (IDIf-04). Similarly, another elderly farmer reports, “The FD officials told me that we can help you in providing plants for free if you can adopt agroforestry which will be of many benefits to you and then …. they were not able to provide me plants of my interest. Therefore, I told them, thank you for helping me, but I am not interested in taking your plants” (IDIf-06). Another elderly man expressed that free distribution of plants as an incentive was too small to influence farmers’ adoption. He reports, *“*There were areas where farmers required fruit plants and poplars; however, the FD was distributing willows and Robinia pseudoacacia. Also, there were areas where farmers required willows and Robinia pseudoacacia however, the FD was distributing Eucalyptus there. In such cases farmers visited the nurseries to obtain plants which showed their intentions to adopt agroforestry however, they returned without taking any plants or taking very minimum plants which showed discouragement of farmers by the FD to adopt agroforestry” (IDIf-02).

Although the free distribution of plants was supposed to be a key feature of BTAP, evidence is mixed on whether this was actually an incentive. This is confirmed by VDC members, as many report that though they did not receive the plants of their choice, they still collected plants and adopted agroforestry. An elderly man who was a member of a VDC and is well respected in the community reports, “Though there was non-availability of fruit plants and a shortage of poplar, however since the FD was providing plants for free, therefore, I told my community to adopt agroforestry” (IDIV-07).

Our findings support Workman et al. (2003), who find that many factors, including farmers’ demographic situations, current knowledge, current skills, existing practices, and farmers’ needs, frame land-use decisions. These also require appropriate analysis before promoting agroforestry. Gomes et al. (2020) identify that assessments of different plant production areas and the vulnerability of different areas to climate change may direct climate adaptation management actions, where adoption of agroforestry systems can mitigate the effects of climate change and maintain 75% of the area suitable for production. These results are also consistent with those of Becot and Inwood (2020). Thus, it is important to note that the mere distribution of free plants should not be considered the final goal in envisioning policies for the diffusion of agroforestry. An intermediate outcome for achieving long-term sustainability in agroforestry diffusion requires careful assessment of farmers’ plants and species needs. This is because if the distributed plants or species are not of community interest, farmers are less likely to accept the plants and, consequently, less likely to adopt agroforestry.

#### Poor quality of distributed plants

The perceptions of both farmers and VDC members are that the distributed plants were of poor quality, thus becoming yet another barrier to agroforestry adoption; see Table 5. Most respondents in both community groups (farmers: 74.33% and VDC members 57.86%) perceived that the low quality of plants distributed in the study area hindered adoption of agroforestry during BTAP.

The results of our in-depth interviews reveal that the quality of distributed plants is critical in motivating farmers to adopt an effective agroforestry system. They stated that sometimes the FD distributed low-quality plants to community members, which discouraged them from adopting agroforestry. In-depth interviews reveal that distributions of poor quality plants discouraged farmers from collecting plants in the first place, sometimes even not taking the free plants despite visiting FD offices. The primary author discussed no personal observations of low-quality plants upon which one of the farmers who was a respected elder in the community reported, “Why would a nursery owner distribute low-quality plants in front of you, any officer, or some persons who are strictly monitoring them. They never will distribute low-quality plants in front of you. However, when you were not here, they used to distribute low-quality plants” (IDIV-01). Similarly, another elderly farmer reports, “I have a pictorial proof of low-quality plants distribution by forest department, which has affected not only me but my entire community adoption decisions. No one was willing to participate in agroforestry. Even the elders have forbidden their community members to participate in the agroforestry program of the BTAP” (IDIV-02). That appears true for many villages, with elderly farmers possessing pictorial and video proof of FD poor-quality plant distribution; the evidence was shown directly to the lead author of this paper.

The quality of distributed plants is a key element for the successful adoption of agroforestry initiatives (Degrande et al. 2013). Those who wish to scale up agroforestry should consider using high-quality plants, which has emerged as a factor affecting farmer willingness to adopt the agroforestry system (Degrande et al. 2013). The results from our focus group discussions also confirm that there was a lack of good-quality plants for distribution in many nurseries, which played a crucial role in the low adoption of agroforestry. This means that the availability of good-quality plants for free distribution should be considered as a greater incentive for farmers to adopt an agroforestry system. Similarly, the benefits from agroforestry adoption can only be reaped when farmers will have access to good-quality plants. In line with this, Dlamini (2020) argues that even if agroforestry is important to people, its adoption will be sustained only when there are good quality free plants available to local communities as an incentive. Our findings confirm the previous results of Do et al. (2020), who report that access to good-quality plants increases people’s participation in the adoption of agroforestry systems and forest conservation activities.

**Fig. 4:**
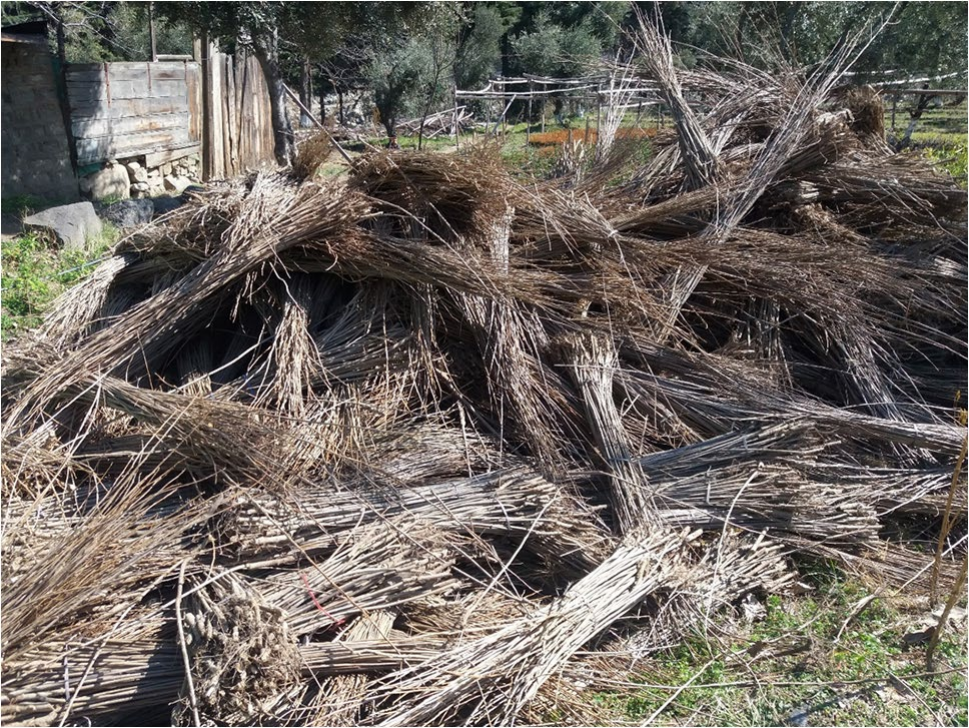
Dried (died) plants have been distributed under agroforestry in 2017

The forest department may not be able to achieve the objective of agroforestry diffusion under BTAP. To achieve objectives of agroforestry diffusion under BTAP, the government must ensure that the distributed plants are of good quality. Dlamini (2020) also suggests that timely adoption of agroforestry can ensure the quality of plants. Prior to plant distribution under agroforestry/farm forestry, programs that promote timely and sustainable agroforestry adoption are important for maintaining the high quality of distributed plants. Policies for timely distribution of plants will help communities and households to collect agroforestry plants before they start drying out and dying (Fig. 4).

#### Poor know-how of effective plantations

Farmers and VDC members both perceive that the poor know-how of effective plantation among farming communities acts as a barrier to agroforestry adoption in the study area; results are presented in Table 5. Most community respondents (farmers: 72.0% and VDC members 54.29%) perceived that the poor know-how of effective plantations among farming communities in the study area hinders farming households from adopting agroforestry under BTAP.

Despite the potential of agroforestry to improve crop yields, energy provision, soil erosion control, and water-use efficiency, farmers in the study region are slow to adopt. Farmers’ awareness may play a role in the widespread adoption of agroforestry. The in-depth interviews report poor knowledge of effective plantation, especially among resource poor farmers. An elderly farmer reports, “In my community, the farmers will agree that agroforestry may improve food and firewood collection and perhaps may increase the income of a household. People are aware that agroforestry improves soil fertility and crop productivity. They know that agroforestry can help in mitigating major risks such as droughts and floods. People have experienced everything in several projects that were involved in promoting agroforestry such as BTAP. However, the community members at large do not have the know-how of keeping plant to plant distance which fails the entire plantations. Farmers don’t know how to remove tubes from the plants. What will be the benefits of such a plantation if a farmer is growing shade trees at the space of 6 inches?” (IDIV-03).

Mulyoutami et al. (2020) report that farmers’ know-how and skills with respect to effective plantation affect agroforestry adoption and management. The lack of technical knowledge among farmers along with non-availability of extension staff results in poor adoption of agroforestry at the local level. Since the lack of technical knowledge is a major barrier for affective plantation and management of plantation sites, policy should be designed to provide training to farmers on plantations and management of plantation sites (Mulyoutami et al. 2020). If farmers are provided appropriate knowledge and training, they will be able to collect quality plants on time and ensure their timely planting and protection in the field. Practical tasks on plantation techniques, tools, and teaching materials will also increase farmers’ know-how of effective plantation and will develop their interest in adopting agroforestry.

#### FD does not keep promises which negatively influence the community trust on them

About 67.0% of farmers and 51.43% of VDC members responding to our interviews report that the FD did not keep promises, thus adversely affecting the adoption of agroforestry under BTAP (Table 5). They report that the FD attitude toward promises made with community members described their agroforestry adoption and management system. However, in most cases, the FD failed to fulfill their promises (Table 5). Our in-depth interviews identify that FD officials visited communities and made promises but hardly fulfilled any. Even on a routine basis the project staff organized community meetings where they gave hope to farmers, but then failed to deliver. During in-depth interviews farmers reveal that, in order for the FD to have their support with project execution, promises must be kept. A respected community elder reports, “People from government organizations come and go. They always come here and talk to us about the future and development of our area. But, after serious discussions and long talks they only say “would be” “we will” “soon” and “we are planning”. However, they do not help our community” (IDIV-03). Similarly, another elderly man reports, “We want to see something implemented from the government side. What is the meaning of spending a long time every day and wasting their and our energy? No implementation from their sides means “false hope.” Now we do not trust them. They may be promoting farm forestry to develop us. However, our development will hardly come with agroforestry. If they want us to develop, they should provide us with alternative energy sources and seal our dependence on firewood for cooking and heating (IDIf-10).

This is similar to the results of Wondirad and Ewnetu (2019) and Leonard (2019), both reporting that the nature of service providers determine the extent of farmers’ trust and participation in a developmental activity with strong trust between project stakeholders and communities improving community engagement in development activity. Thus, the policy should be that the VDC only take promises from the FD on those activities that the FD can fulfill so that they can keep the trust of their communities. Furthermore, the FD should be made aware of the consequences of making promises that they cannot fulfill. While planning and implementing agroforestry activities, the FD should only make those promises that they can fulfill and should not make promises that they cannot fulfill. Similar policies are suggested by Ullah et al. (2022) for promoting landscape restoration in the study region.

#### Wastage of plants

The results of our in-depth interviews reveal that 51.0% of farmers and 43.57% of VDC members responding to our interviews report that the community members wasted too many plants, thus adversely affecting the FD’s distribution of agroforestry (Table 5). During in-depth interviews with VDC members, reports indicate that farmers took more plants than they could grow. An elderly farmer who is a member of a VDC reports, “I observed many people throwing plants into rivers and in dusts” (IDIV-10). Many community members (farmers) also agreed to the statements of elderly people (VDC members). A farmer reports, “Since I don’t trust government work, I took more plants from the FD” (IDIf-07). Similarly, another farmer reports, “I had place for 400 plants but I took 1000 plants from the FD. Out of 1000, the 400 plants were of extremely good quality and therefore, I planted them” (IDIf-10). Another farmer report*s*, “I thought I was getting these plants for free anyway. Why not get more plants. I can throw away the useless, dry and small ones and can grow the healthy ones” (IDIf-03).

There should be a policy to check the availability of space with farming communities to ensure that the farmers have a place for the number of plants they collect. The VDC should keep eyes on farmers who collect more plants than their capacity. Such policies will prevent the waste of time and will develop trust among community members, the VDCs, and the FD, thus, ultimately, increasing agroforestry adoption.

## Conclusion and policy implications

The findings of our study reveal that not only does the community recognize the need and importance of agroforestry adoption, but it is also aware of climate risks and the role of agroforestry in risk mitigation. The important factors that positively affect agroforestry diffusion include locations of crop fields along riverbanks, communities’ dependency on firewood, and the timber value of agroforestry. Similarly, household-level factors, like age, access to information, and crop cover positively affect farmer adoption of agroforestry under BTAP, while forest cover negatively affects it. At the community level, several factors, like false information by farmers to monitoring teams, non-availability of extension staff, misunderstanding/miscommunication among project staff and community, as well as the lack of sufficient budget for activities affects the FD ability to disseminate agroforestry. Similarly, barriers to communities that prevent adoption of agroforestry included the lack of participation by VDCs in the planning and monitoring of agroforestry program, as well as a lack of plant needs assessments by the project staff, poor quality of plants distributed by the FD, farmers’ poor know-how of effective plantations, plant wastage by the farming community, and the lack of trust among community and project staff.

The findings carry policy implications for making effective agroforestry adoption strategies under the newly launched 10-BTAP to increase the success of the agroforestry system and sustainable forest management. Accordingly, policy-makers and project designers need to pay utmost attention to those factors that have hindered the adoption of agroforestry. For this, high exposure to extension services and increased frequency of extension-farmer contacts to mitigate future climate risks through agroforestry adoption is a necessary condition. The findings suggest the importance of communicating project objectives to the community members and their participation in project planning and execution for making community-focused projects successful in developing countries. The study also shows that community-level agroforestry adoption could be successful by conducting farmers’ needs assessments and restoring farmers’ confidence. As the limited capacity of farmers plays a role in shaping the limited adoption of the agroforestry system and poor success at the community level, it is necessary to focus on strengthening the capacity of farmers. This could include involving the community through extension projects on farm-level plantation awareness campaigns and building their capacity through community engagement and climate finance readiness. Finally, for effective diffusion of agroforestry, the FD should focus on farming communities that reside near crop fields as compared to farming communities that reside on the margins of forests.

## Supplementary Information

Below is the link to the electronic supplementary material.

Supplementary file1 (DOCX 13 KB)

## Data Availability

The authors confirm that the data supporting the findings of this study will be available from the corresponding author on reasonable request.
