# Supplementary material for: Constraints to agroforestry diffusion under the Billion Trees Afforestation Project (BTAP), Pakistan: policy recommendations for 10-BTAP
Source: Environ Sci Pollut Res Int. 2022 May 12;29(45):68757–75. doi: 10.1007/s11356-022-20661-9 (PMC9508197; doi:10.1007/s11356-022-20661-9)
Supplement: Supplementary file 1 — Supplementary file1 (DOCX 13 KB) [file 11356_2022_20661_MOESM1_ESM.docx]

Table 1. Results of multicollinearity test.

| Variables | Coefficients | | | | | | |
| --- | --- | --- | --- | --- | --- | --- | --- |
|  | Unstandardized Coefficients | | Standardized Coefficients | t | Sig. | Collinearity Statistics | |
|  | B | Std. Error | Beta |  |  | Tolerance | VIF |
| Age | .006 | .001 | .154 | 6.021 | .000 | .753 | 1.328 |
| Education | -.003 | .002 | -.025 | -1.117 | .265 | .976 | 1.025 |
| Farm size | -.008 | .006 | -.045 | -1.323 | .187 | .429 | 2.333 |
| Family size | -.007 | .003 | -.062 | -2.157 | .032 | .599 | 1.670 |
| Dependence on firewood for cooking and heating | .264 | .037 | .264 | 7.184 | .000 | .366 | 2.732 |
| Access to information | .139 | .027 | .134 | 5.198 | .000 | .741 | 1.349 |
| Forest cover | -.215 | .028 | -.213 | -7.797 | .000 | .666 | 1.501 |
| Crop cover | .090 | .028 | .083 | 3.220 | .001 | .739 | 1.354 |
| Negligence of a farmer by a forest employee | -.323 | .032 | -.314 | -10.110 | .000 | .514 | 1.945 |
| Ownership of animals | .042 | .024 | .041 | 1.795 | .074 | .965 | 1.036 |
